# Cellulose and protein nanofibrils: Singular biobased nanostructures for the design of sustainable advanced materials

**DOI:** 10.3389/fbioe.2022.1059097

**Published:** 2022-12-13

**Authors:** Ana C. Q. Silva, Armando J. D. Silvestre, Carla Vilela, Carmen S. R. Freire

**Affiliations:** Department of Chemistry, CICECO—Aveiro Institute of Materials, University of Aveiro, Aveiro, Portugal

**Keywords:** cellulose, proteins, nanofibrils, nanostructured materials, advanced materials, sustainability

## Abstract

Polysaccharides and proteins are extensively used for the design of advanced sustainable materials. Owing to the high aspect ratio and specific surface area, ease of modification, high mechanical strength and thermal stability, renewability, and biodegradability, biopolymeric nanofibrils are gaining growing popularity amongst the catalog of nanostructures exploited in a panoply of fields. These include the nanocomposites, paper and packaging, environmental remediation, electronics, energy, and biomedical applications. In this review, recent trends on the use of cellulose and protein nanofibrils as versatile substrates for the design of high-performance nanomaterials are assessed. A concise description of the preparation methodologies and characteristics of cellulosic nanofibrils, namely nanofibrillated cellulose (NFC), bacterial nanocellulose (BNC), and protein nanofibrils is presented. Furthermore, the use of these nanofibrils in the production of sustainable materials, such as membranes, films, and patches, amongst others, as well as their major domains of application, are briefly described, with focus on the works carried out at the BioPol4Fun Research Group (Innovation in BioPolymer based Functional Materials and Bioactive Compounds) from the Portuguese associate laboratory CICECO–Aveiro Institute of Materials (University of Aveiro). The potential for partnership between both types of nanofibrils in advanced material development is also reviewed. Finally, the critical challenges and opportunities for these biobased nanostructures for the development of functional materials are addressed.

## Introduction

The transition towards a more sustainable society, aligned with the 2030 Agenda for Sustainable Development (United Nations, 2015), demands a considerable change in the overall design and manufacturing practices, regarding consumer items and materials with advanced functionalities (Silva et al., 2021b). In this perspective, naturally abundant and renewable biobased feedstocks are increasingly being exploited as eco-friendly building blocks for the development of sustainable materials with diverse morphologies and applications (Silva et al., 2014a; Silva et al., 2021b; Shaghaleh et al., 2018; Mohammadian et al., 2020; Shen et al., 2021; Teixeira et al., 2022a).

The advent of nanotechnology and nanoscience has created an opportunity to control materials at the nanoscale, enabling the tailored design of complex nanostructures such as nanotubes, nanowires, nanofibrils, and nanoparticles (Krishnaswamy and Orsat, 2017; Carvalho et al., 2021b). Nanofibrils, i.e., fibers with diameter in the nanoscale and significantly longer lengths (up to several micrometers), have attracted the interest of researchers as essential building blocks in the development of innovative functional materials, owing to their outstanding mechanical properties (Barhoum et al., 2018; Barhoum et al., 2019). Particularly, nanofibrils obtained from polysaccharides (e.g., cellulose, chitosan/chitin) (Raza et al., 2021) and proteins (e.g., silk fibroin, soy protein, zein) (Babitha et al., 2017) combine the unique features of nanofibrils with biocompatibility, biodegradability, and in some cases specific biological properties, that can be exploited for the preparation of functional materials, such as membranes (Galiano et al., 2018), hydrogels (Ling et al., 2018b; Du et al., 2019), and films (Lendel and Solin, 2021), among others (Knowles and Mezzenga, 2016; Owolabi et al., 2020; Santos et al., 2021). These nanomaterials find application in high-tech applications, as for example, in enzyme immobilization (Frazão et al., 2014), functional textiles (Silva et al., 2021a), active food packaging (Cazón and Vázquez, 2021; Daniloski et al., 2021), tissue engineering and wound healing (Carvalho et al., 2019; Chen et al., 2022; Soroush et al., 2022), drug delivery (Almeida et al., 2014; Silvestre et al., 2014), cosmetic applications (Almeida et al., 2021; Meftahi et al., 2022), sensors and semiconductors (Cherian et al., 2022; Qiu et al., 2022), fuel cells (Vilela et al., 2019e), and environmental remediation (Muqeet et al., 2020; Abdelhamid and Mathew, 2021; Peydayesh and Mezzenga, 2021; Raza et al., 2021).

As the most abundant polysaccharide on Earth, cellulose has attracted increased scientific and economic interest in materials development (Charreau et al., 2020; Meftahi et al., 2021), particularly in its nanometric forms, *viz.* cellulose nanocrystals (CNCs), nanofibrillated cellulose (NFC) and bacterial nanocellulose (BNC) (Freire et al., 2013; Figueiredo et al., 2014; Freire and Vilela, 2022). The majority of literature in this field addresses both established and novel methods for isolating and modifying nanocelluloses (Abdul Khalil et al., 2012; Meftahi et al., 2021; Sayyed et al., 2021; Hamimed et al., 2022; Pradhan et al., 2022) and their application in materials science (Carvalho et al., 2019; Vilela et al., 2019e; Wang et al., 2020; Subhedar et al., 2021; Wang et al., 2022). Thus, NFC and BNC will be the focus of this review concerning cellulose nanofibrils application in this field.

On the other hand, owing to the recent advances in protein fibrillation mechanisms and methodologies (Wang et al., 2021), protein nanofibrils (also known as amyloid fibrils) obtained from both animal and plant-based origins (Meng et al., 2022) have stepped forward as promising nanostructures for the development of materials with a variety of functional properties that are gaining attention for packaging purposes (Daniloski et al., 2021; Hadidi et al., 2022), environmental remediation (Peydayesh and Mezzenga, 2021; Vinayagam et al., 2022), and biomedical applications (Silva et al., 2014a; Mohammadian et al., 2020), amongst others.

The production, modification, and applications of cellulose and protein nanofibrils have already been extensively reviewed, as proven by the number of review articles and book chapters supra cited. Nevertheless, as far as our research could go, only one review by Ling et al. (2018a) provided an overview of both polysaccharide and protein-based nanofibrils, specifically cellulose, chitin, silk, and collagen nanofibrils, for material production. This comprehensive work provides a detailed outline of the four most abundant biopolymer nanofibrils in terms of structure, computational models, processing methodologies, and applications. Nonetheless, the authors only offer information regarding fibrous proteins (i.e., silk and collagen) despite the growing interest in the fibrillation process of globular proteins [e.g., β-lactoglobulin (Loveday et al., 2017), lysozyme (Mohammadian and Madadlou, 2018)] with more complex structural levels and essential biological properties.

The present review will focus on nanocellulose fibrils, namely NFC and BNC, and protein amyloid nanofibrils, to design advanced and sustainable materials. Though cellulose nanocrystals (CNCs) are isolated from cellulosic feedstocks, the resulting highly-crystalline rod-like nanostructures contain all three exterior dimensions at the nanoscale and are classified as cellulose nanoparticles (Barhoum et al., 2019). As a result, this cellulose nanoform is beyond the scope of this review. Nonetheless, the reader can find more information about recent advances in the preparation and modification of CNCs, material fabrication, and significant application areas elsewhere (Long et al., 2021; Rana et al., 2021; Shojaeiarani et al., 2021).

**FIGURE 1:**
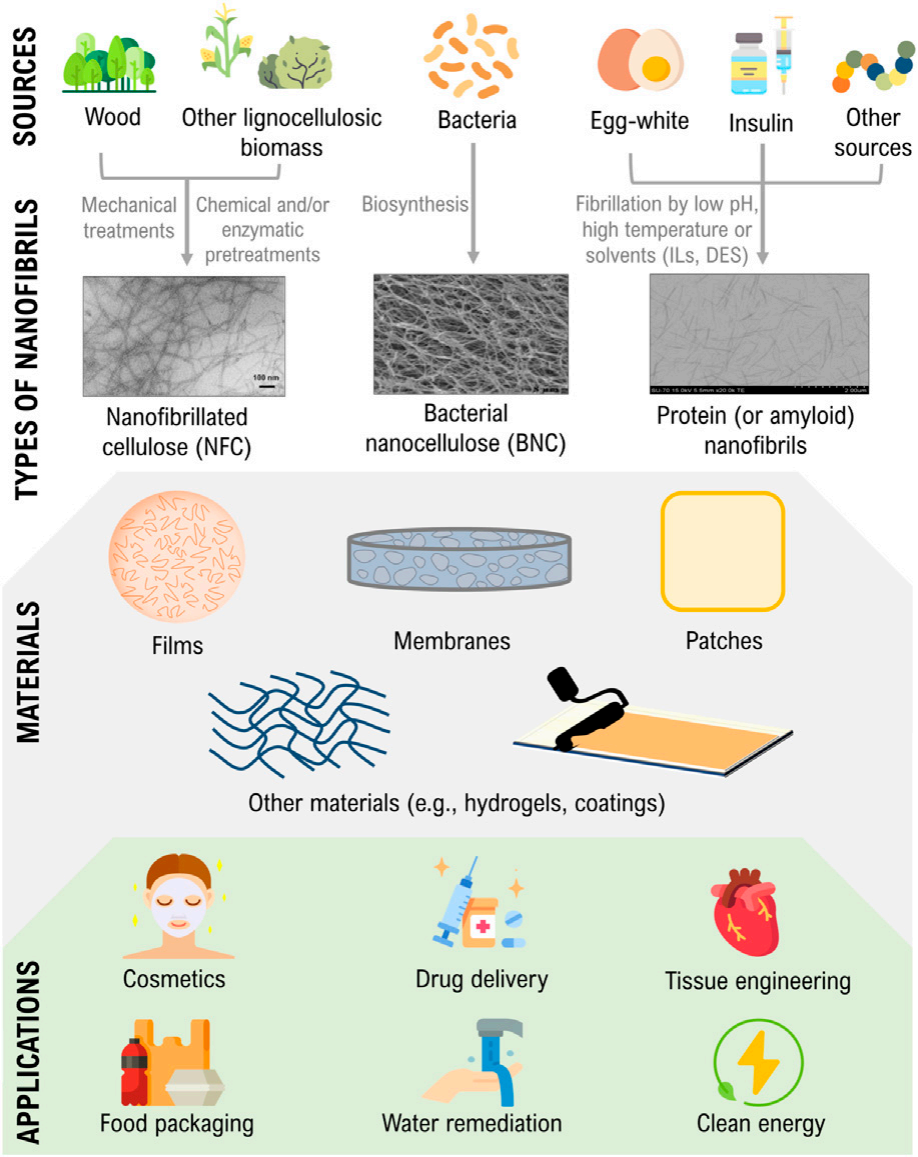
Illustration of the sources and production of nanocellulose and protein nanofibrils, their use in materials design, and critical areas of application. Transmission electron microscopy (TEM) micrographs are adapted and reprinted with permission from (Nazrin et al., 2020). Copyright Frontiers, 2020; and (Silva et al., 2018c). Copyright Elsevier, 2018.

Thus, this review provides a succinct overview regarding the fabrication methodologies and properties of nanocellulose and protein nanofibrils, their use in materials design, and critical areas of application (Figure 1), with emphasis on the works developed in the latter years at the BioPol4Fun Research Group (Innovation in BioPolymer based Functional Materials and Bioactive Compounds) from the Portuguese associate laboratory CICECO—Aveiro Institute of Materials (University of Aveiro). The combination of both types of nanofibrils is also examined before concluding with some prospects for the exploitation of these nanostructures for materials research. Since it is impossible to dive into the detail of every cited work, a few key examples and features of representative materials have been chosen for a more elaborated discussion in the following sections. Further information concerning the type, composition, fabrication methodologies, and critical properties and applications of each material is summarized on Tables 1–5.

**TABLE 1:** Examples of membranes produced with cellulosic nanofibrils.

Type of nanofibrils	Other compounds	Methodology	Key properties and applications	Reference(s)
BNC	APS	Chemical grafting	Antibacterial activity: *E. coli*; *S. aureus*Non-cytotoxic (adipose-derived stem cells)	Fernandes et al. (2013)
BNC	APS, AEAPS	Chemical grafting	Antibacterial activity: *S. aureus*	Chantereau et al. (2019a)
BNC	Fucoidan (0.5 and 0.75)^a^	Diffusion of aqueous solutions in the BNC matrix	Ion-exchange membranes for fuel cells	Vilela et al. (2020c)
			Maximum ionic conductivity: 1.6 mS cm^−1^	
BNC	Lignosulfonates (0.5 and 0.75)^a^	Diffusion of aqueous solutions in the BNC matrix	Ion-exchange membranes for fuel cells	Vilela et al. (2020b)
			Maximum ionic conductivity: 23 mS cm^−1^	
BNC	Nafion^®^ (0.5)^a^	Diffusion of the ionomer in the BNC matrix	Ion-exchange membranes for fuel cells	Gadim et al. (2016)
			Maximum ionic conductivity: 140 mS cm^−1^	
BNC	P(bis-MEP) (3 and 5)^a^	*in situ* free radical polymerization	Ion-exchange membranes for fuel cells	Vilela et al. (2018b)
			Maximum ionic conductivity: 30 mS cm^−1^	
BNC	PAEM (6)^a^	*in situ* free radical polymerization	Antibacterial activity: *E. coli*	Figueiredo et al. (2015a)
			Improvement of the thermal stability and water uptake capacity	
BNC	PGMA (2.0 and 20.0)^a^	*in situ* free radical polymerization	Improvement of the thermal stability and water uptake capacity	Faria et al. (2019a); Faria et al. (2019b)
			Decrease in hydrophobicity	
BNC	PMACC (2, 5, 8 and 10)^a^	*in situ* free radical polymerization	Ion-exchange membranes for fuel cells	Vilela et al. (2017)
			Maximum ionic conductivity: 10 mS cm^−1^	
BNC	PMMA, PBA	*in situ* atom transfer radical polymerization	Improvement of the thermal stability	Lacerda et al. (2013)
			Increased hydrophobicity	
BNC	PMOEP (3, 5 and 10)^a^	*in situ* free radical polymerization	Ion-exchange membranes for fuel cells	Vilela et al. (2016)
			Maximum ionic conductivity: 100 mS cm^−1^	
BNC	PMPC (3 and 5)^a^	*in situ* free radical polymerization	Antibacterial activity: *E. coli; S. aureus*	Vilela et al. (2019b)
			Water remediation (removal of cationic and anionic organic dyes)	
BNC	PSSA (5)^a^	*in situ* free radical polymerization	Ion-exchange membranes for fuel cells	Gadim et al. (2014); Gadim et al. (2017); Vilela et al. (2020a)
			Maximum ionic conductivity: 185 mS cm^−1^ Application in a microbial fuel cell with *Shewanella frigidimarina*	

^a^ Nominal composition represented as the mass ratio of other compounds in relation to the nanofibrils $\left(W_{other\;compounds}/W_{nanofibrils}\right)$ .

Abbreviations: AEAPS, (2-aminoethyl)-3-aminopropyl-trimethoxysilane; APS, 3-aminopropyl-trimethoxysilane; BNC, bacterial nanocellulose; P(bis-MEP), poly(bis[2-(methacryloyloxy)ethyl] phosphate); PAEM, poly(2-aminoethyl methacrylate); PBA, poly(butyl acrylate); PGMA, poly(glycidyl methacrylate); PMACC, poly(methacroylcholine chloride); PMMA, poly(methyl methacrylate); PMOEP, poly(methacryloyloxyethyl phosphate); PMPC, poly(2-methacryloyloxyethyl phosphorylcholine); PSSA, poly(4-styrene sulfonic acid).

## Exploiting nanocellulose fibrils for the production of novel materials

**FIGURE 2:**
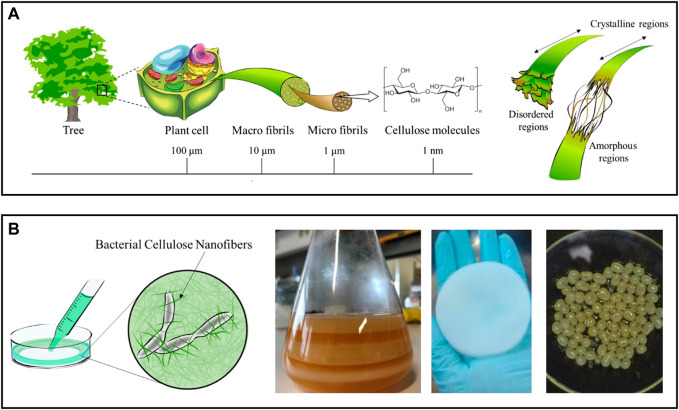
**(A)** Schematic representation of the hierarchical structure of cellulose obtained from plants, with detail in the crystalline and amorphous regions of the cellulose nanofibrils. Adapted and reprinted with permission from (Miyashiro et al., 2020). Copyright MDPI, 2020; **(B)** Diagram of a bacterial nanocellulose culture and digital photographs of membranes produced in static culture before (left) and after cleaning (center), and of spherical particles obtained in agitated culture conditions (right). Adapted and reprinted with permission from (Miyashiro et al., 2020). Copyright MDPI, 2020; and (Almeida et al., 2021). Copyright MDPI, 2021.

With an estimated annual production of 10^11^–10^12^ tonnes, cellulose is considered the most prevalent natural polymer and can be extracted from many sources, mostly from plants (lignocellulosic biomass), but also from algae, bacteria, and tunicates (Nechyporchuk et al., 2016; Trache et al., 2020). This linear polysaccharide is comprised of D-glucose units linked *via* β-(1→4) glycosidic bonds. Due to the strong intra- and intermolecular hydrogen bond network and the intermolecular van der Waals forces, the cellulose chains arrange themselves into a distinctive three-dimensional structure of microfibrils that contain crystalline (highly ordered) and amorphous domains (Figure 2A) (Noremylia et al., 2022). The intertwining of microfibrils culminates in the formation of macrofibrils.

As overviewed by Klemm et al. (2018), Thomas et al. (2020) and, more recently, by Noremylia et al. (2022), nanocelluloses can be extracted from native cellulose using top-down approaches, or obtained by bottom-up methodologies, producing nanostructures with distinct morphological features. According to their characteristics, three types of nanocellulose can be identified: 1) Cellulose nanocrystals (CNCs), also known as “nanocrystalline cellulose” or “cellulose nanowhiskers,” 2) Nanofibrillated cellulose (NFC), also commonly referred to as “microfibrillated cellulose” or “cellulose nanofibrils,” and 3) Bacterial nanocellulose (BNC), also denominated “microbial cellulose,” “biocellulose” or “biotech cellulose” (Klemm et al., 2018). As previously stated, this section will provide a brief rundown of the fabrication processes and attributes of NFC and BNC. Regardless, the reader is encouraged to examine the publications mentioned in each section, which provide a comprehensive overview of these topics.

Plant cellulose can be used to obtain nanofibrils (NFC) with a diameter of 5–60 nm and a length of a few micrometers, which contain both amorphous and crystalline regions (Figure 2A) (Nechyporchuk et al., 2016). Delamination of cellulose fibers is most frequently accomplished in an aqueous medium through intense mechanical treatments (e.g., high-pressure homogenization, grinding, and refining), resulting in suspensions of NFC with low solid content (<5 wt%) (Nechyporchuk et al., 2016; Noremylia et al., 2022). Despite the high energy consumption associated with these processes, this is the most scalable methodology and, as a result, the preferred option for industrial applications. Nonetheless, chemical and enzymatic pretreatments are also proposed to minimize the energy consumption in mechanical processing, while also increasing the degree of fibrillation of NFC, as these pretreatments disturb the hydrogen bond network (Pradhan et al., 2022; Squinca et al., 2022). Nevertheless, depending on the selection and combination of pretreatments and extraction processes, various grades of NFC with variable dimensions and properties (e.g., crystallinity, rheological behavior, surface chemistry) can be obtained (Nechyporchuk et al., 2016). Non-traditional processes, such as extrusion, ball milling, steam explosion, aqueous counter collision, cryocrushing, and ultrasonication, have been proposed to govern the fibrillation process yielding NFC with more predictable properties (Wang et al., 2021).

In contrast, BNC results from a bottom-up process in which non-pathogenic bacterial strains synthesize the nanofibrils through a sequence of biochemical reactions driven by specific enzymes and cofactors (Manan et al., 2022). Most common BNC-producing species belong to the *Komagataeibacter* genus (previously known as *Gluconacetobacter* (Trovatti et al., 2011a)), although the list may include organisms from the *Agrobacterium*, *Rhizobium*, and *Pseudomonas* genus. In practice, BNC is a three-dimensional nanofiber network resulting from a fermentation process taking place in a nutrient-enriched culture medium (Klemm et al., 2018). Depending on the culture conditions, this network can be produced with tailored shapes and sizes, ranging from planar hydrogel pellicles, formed at the air-water interface of the culture media in static cultures, to sphere-like particles in agitated cultures (Figure 2B) (Chandana et al., 2022). The use of bioreactor systems with complex geometries is known to improve BNC yield, enabling the production of BNC on a larger scale (Chandana et al., 2022). Compared to NFC, bacterial nanocellulose has a high degree of crystallinity because it is free of hemicelluloses, lignin, and other minor compounds found in plant-lignocellulosic biomass (Klemm et al., 2018). BNC also has an exceptional water retention ability since it contains nearly 99% water.

Overall, cellulosic nanofibrils have remarkable properties, such as high surface area, high aspect ratio, tailorable surface chemistry, high mechanical strength, rheological behavior, high water absorption capacity, non-cytotoxicity, non-genotoxicity, and inherent renewability (Chandana et al., 2022; Noremylia et al., 2022), which elevate this polysaccharide out of the shadows of the pervasive pulp and paper industries to produce sophisticated materials, with diverse types and functionalities. However, due to the presence of three hydroxyl groups per anhydroglucose unit, these nanofibers are extremely hydrophilic, which can be seen as a benefit in applications in which water compatibility is an advantage (e.g., most often in biomedical applications) or as a drawback when compatibilization with hydrophobic domains is essential, thus requiring adequate modifications towards their hydrophobization.

The following sections illustrate some of the most recent and relevant contributions about the use of NFC and BNC to assemble membranes, films, patches and other materials.

### Membranes

The simplicity of creating membranes with tailored size and shape that do not disintegrate when exposed to water-rich environments is a particular advantage of BNC materials in several sectors. NFC-based membranes can also be produced by simple methodologies, like casting or filtration (Table 1). Moreover, cellulose nanofibrils have a high surface area and are simple to modify. As such, the properties of BNC and NFC can be customized to accomplish specific functions (Shen et al., 2020). For instance, the chemical grafting of amino moieties grants antibacterial activity to BNC fibrils (Fernandes et al., 2013; Chantereau et al., 2019a), and the *in situ* atom transfer radical polymerization reaction of acrylate monomers onto the BNC nanofibrils surface can improve the hydrophobicity of the membranes (Lacerda et al., 2013). Additionally, the *in situ* free radical polymerization of acrylate monomers inside the 3D-network of BNC can increase their water uptake capacity (Figueiredo et al., 2015a), depending on the starting monomers and conditions. To illustrate, the grafting of poly(methyl methacrylate) in the BNC network increased the water static contact angle of the membrane from 32° up to 134°, thus enhancing its hydrophobic behavior (Lacerda et al., 2013). Owing to the properties mentioned above, allied with their tunable porous structure, nanocellulose-based materials are receiving significant attention for the fabrication of high-performance membranes for environmental remediation purposes, with application as ion-selective separators for clean energy, in water purification, air filtration, and carbon dioxide sieving (Wang et al., 2022).

In the energy field, nanocellulose membranes are being exploited to design components of energy storage systems, in the development of electrodes *via* direct carbonization of the cellulose nanofibrils or incorporation of conductive polymers (e.g., polypyrrole), carbon (e.g., carbon nanotubes) and metal/metal oxides (e.g., silver, manganese dioxide) phases (Xiao et al., 2022). Nanocelluloses are also being explored as alternatives for components in energy generators like fuel cells, particularly as replacements for the ion-exchange membranes (Vilela et al., 2019e). Nanocelluloses do not naturally possess the ionic conductivity required for this application; however, this problem can be solved by incorporating ion-conducting phases either through the direct diffusion of ionomers, like Nafion^®^, into the BNC 3D-nanofibrillar structure (Gadim et al., 2016) or *via* the *in situ* free-radical polymerization under green conditions of adequate synthetic monomers to generate polyelectrolytes, such as poly(4-styrene sulfonic acid) (Gadim et al., 2014, 2017; Vilela et al., 2020a), poly(methacryloyloxyethyl phosphate) (Vilela et al., 2016), poly(methacroylcholine chloride) (Vilela et al., 2017) and poly(bis[2-(methacryloyloxy)ethyl] phosphate) (Vilela et al., 2018b). More recently, BNC and naturally derived macromolecules rich in ion-conducting functional groups have been combined to create fully biobased ion-exchange membranes. Fucoidan, an algal polysaccharide (Vilela et al., 2020c), and lignosulfonates, a by-product of the sulfite pulping process (Vilela et al., 2020b), are examples of this practice. Despite the ionic conductivity of some of these systems being two orders of magnitude lower than Nafion^®^, the benchmark for this application, these findings show the immense potential of cellulose nanofibrils for creating environmentally friendly separators.

Sorbents of modified nanocellulose membranes have been described to remove heavy metal ions, dyes, pesticides, pharmaceuticals, and other dissolved organic pollutants from contaminated waters (Muqeet et al., 2020; Abdelhamid and Mathew, 2021). Since cellulosic nanofibrils have low affinity for ionic species, it is necessary their combination with materials with high adsorption capacity (e.g., graphene oxide) (Abdelhamid and Mathew, 2021) or modification with adequate functional groups (e.g., carboxylate or quaternary ammonium moieties) to which metal ions, dyes or other contaminants (e.g., sulfate and fluoride anions) can bind to. Functional groups can also be imparted to cellulose nanofibers during the production stages, in the case of NFC, or by post-modification (Abdelhamid and Mathew, 2021). A common example is the introduction of carboxyl groups (COO⁻) *via* TEMPO-mediated oxidation of the C6 primary hydroxyl group, which is often used in the nanocellulose production stages. Cellulose nanofibrils with anionic functional groups (e.g., COO⁻) ensure high adsorption toward cationic contaminants. In contrast, nanofibrils bearing cationic groups can be effective sorbents for anionic compounds (e.g., fluoride) (Abdelhamid and Mathew, 2021).

**FIGURE 3:**
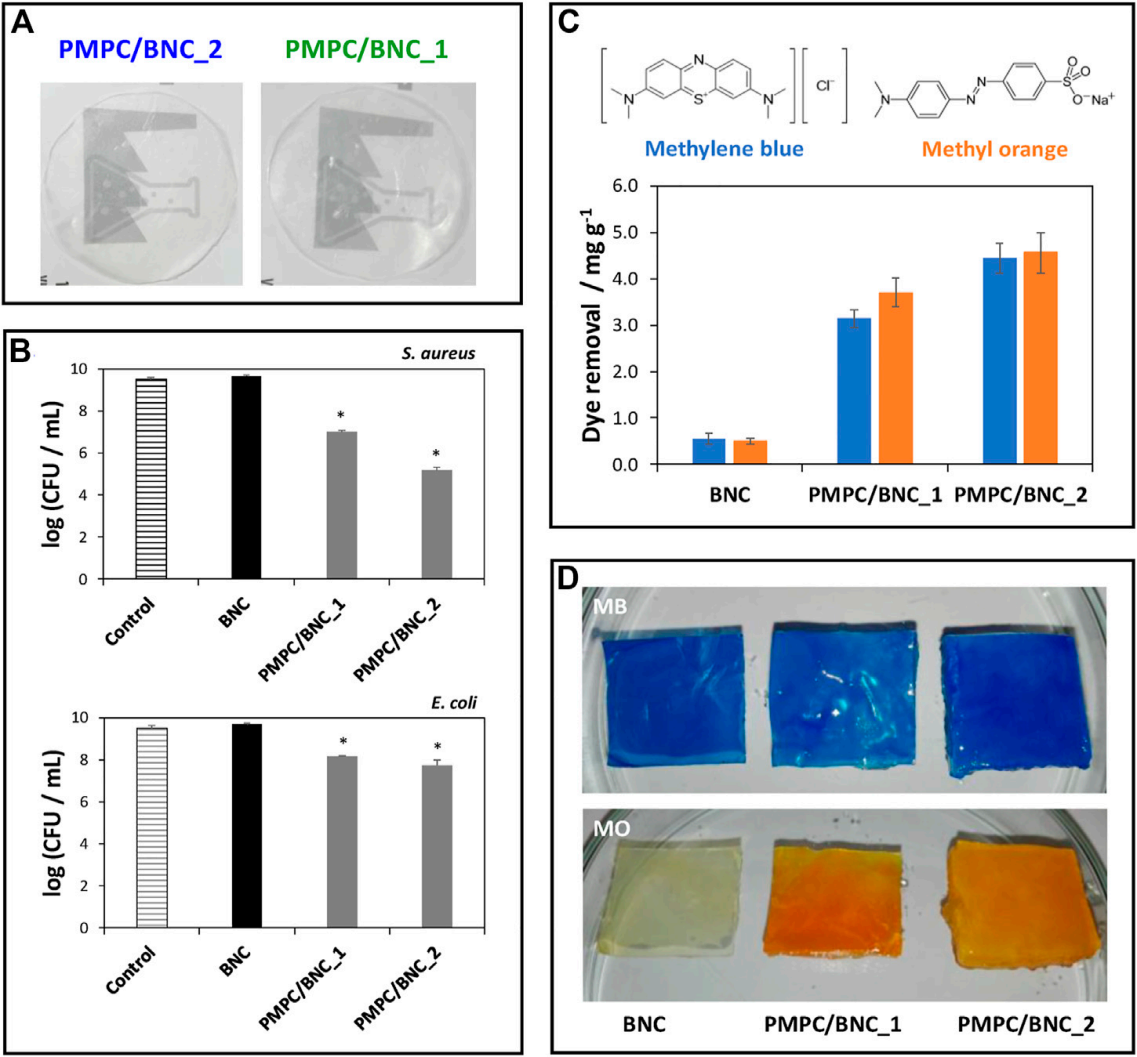
**(A)** Digital photographs of the zwitterionic membranes; **(B)** Graphical representation of the antibacterial activity of the membranes against *S. aureus* and *E. coli* after 24 h of exposure; **(C)** Chemical structures of the methylene blue (MB) and methylene orange (MO) dyes and graphical representation of the dye removal capacity of the BNC membranes with different compositions; **(D)** Digital photographs of the membranes after immersion in the aqueous dye solutions for 12 h. Reprinted with permission from (Vilela et al., 2019b). Copyright MDPI, 2019.

However, the affinity towards both positively and negatively charged molecules is highly desirable in this field. To address that challenge, cellulose nanofibrils can be combined with zwitterionic polymers, such as the non-toxic poly(2-methacryloyloxyethyl phosphorylcholine), that contains a phosphate anion and a trimethylammonium cation (Vilela et al., 2019b). It was shown that this BNC/zwitterionic polymer membrane effectively collected cationic (methylene blue) and anionic (methylene orange) model dyes from contaminated water (ca. 4.4–4.5 mg g^−1^) and limited the growth of pathogens commonly observed in these environments (up to 4.3- and 1.8-log CFU reduction for *Staphylococcus aureus* and *Escherichia coli,* respectively), owing to the antimicrobial action of the polymer, highlighting the effective dual action of the biosorbents in the retrieval of contaminants (Figure 3). Overall, reusable nanocellulose membranes hold the potential for contaminant removal and salvage and repurposing of valuable waste matter, such as metals.

### Films

The fundamental shift towards a more environmentally conscious society has increased the demand for packaging materials made from sustainable resources to reduce the volume and impact of the typical petroleum-based plastics we are accustomed to (Silva et al., 2021b) (Table 2). The inclusion of cellulose nanofibrils in polymeric films results in improved thermal and mechanical properties that are often vital for their target applications (Amara et al., 2021; Cazón and Vázquez, 2021). For instance, both BNC (Trovatti et al., 2012a) and NFC (Trovatti et al., 2012b) have been employed as reinforcing elements in homogeneous and translucent pullulan films *via* the simple solvent casting of aqueous suspensions, leading to nanocomposites films with improved thermal (increase in the maximum degradation temperature, compared to the neat pullulan films) and mechanical (e.g., a 22 times increase in the Young’s modulus value with the inclusion of 10% of BNC, compared to unfilled pullulan matrices) properties.

**TABLE 2:** Examples of films produced with cellulosic nanofibrils.

Type of nanofibrils	Other compounds	Methodology	Key properties and applications	Reference(s)
BNC	PEDOT:PSS	Ink-jet printing	Reduced impedance and the 1/f^2^ noiseApplication in sensors (glioma cells)	Inácio et al. (2020)
BNC	PSBMA (3 and 5)^a^	*in situ* free radical polymerization	UV-light barrier function	Vilela et al. (2019c)
			Antibacterial activity: *E. coli; S. aureus*Maximum ionic conductivity: 1.5 mS cm^−1^Application in active food packaging	
BNC (0, 0.5, 0.1, 0.2, 0.4 and 0.6)^b^	Pullulan	Solvent casting	Improvement of the thermal stability and mechanical properties	Trovatti et al. (2012a)
NFC (0.05, 0.10, 0.25, 0.50 and 0.75)^b^	Arabinoxylans, ferulic acid, or feruloylated arabinoxylo-oligosaccharides	Solvent casting	UV-light barrier function	Moreirinha et al. (2020)
			Antibacterial activity: *E. coli; S. aureus* Antifungal activity: *C. albicans*	
			Antioxidant activity: ca. 90%, DPPH assay	
			Application in active food packaging	
NFC	Mango leaf extract (0.1, 0.2, and 0.3)^a^	Supercritical solvent impregnation	UV-light barrier function	Bastante et al. (2021)
			Antibacterial activity: *E. coli; S. aureus*	
			Antioxidant activity: ca. 84%, DPPH assay	
			Application in active food packaging	
NFC (0, 0.5,0.1, 0.2, 0.4 and 0.6)^b^	Pullulan	Solvent casting	Improvement of the thermal stability and mechanical properties	Trovatti et al. (2012b)

^a^ Nominal composition represented as the mass ratio of other compounds in relation to the nanofibrils $\left(W_{other\;compounds}/W_{nanofibrils}\right)$ .

^b^ Nominal composition represented as the mass ratio of nanofibrils in relation to other compounds $\left(W_{nanofibrils}/W_{other\;compounds}\right)$ .

Abbreviations: BNC, bacterial nanocellulose; DPPH, 2,2-diphenyl-1-picrylhydrazyl; NFC, nanofibrillated cellulose; PEDOT:PSS, poly(3,4-ethylenedioxythiophene):polystyrene sulfonate; PSBMA, poly(sulfobetaine methacrylate); UV, ultraviolet.

Films that also offer active and intelligent functions are becoming ever more relevant in the food industry sector, as packaging acts as both a method of transportation and a mean of preserving the food contained within (Vilela et al., 2018a; Carvalho et al., 2021a). In this sense, films that incorporate additives with antimicrobial (e.g., metal nanoparticles, chitosan), antioxidant (e.g., plant extracts, phenolic compounds), and gas scavenging (e.g., metal oxides) functions can extend the shelf life of packed products, resulting in less food spoilage and waste (Vilela et al., 2018a). As an illustrative example, NFC-based films containing arabinoxylans obtained from brewers’ spent grains and ferulic acid or feruloylated arabinoxylo-oligosaccharides displayed good antioxidant activity (ca. 90%, assessed by the DPPH radical scavenging activity), which is essential to control the oxidative reactions and maintain the sensory properties of the foodstuff (Moreirinha et al., 2020). Furthermore, these films showed antimicrobial activity toward common food pathogens, i.e., Gram-positive (*S. aureus*) and Gram-negative (*E. coli*) bacteria and fungi (*Candida albicans*). The inclusion of these active compounds also offers the opportunity to improve the UV-light properties (decrease in the transmittance values of the films in the UVC (100–280 nm), UVB (280–320 nm) and UVA (320–400 nm) regions) and gas barrier of the films, while retaining the mechanical strength (Young’s modulus up to 7.5 GPa) and flexibility characteristic of the cellulose nanofibrils (Moreirinha et al., 2020). NFC has also been combined with a mango leaf extract using conventional solvent-casting methodologies and alternatively by supercritical solvent impregnation (Bastante et al., 2021). The ensuing films displayed good antioxidant (ca. 84%, using the DPPH assay) and antimicrobial activity (ca. 37% and 91% growth inhibition of *S. aureus* and *E. coli,* correspondingly), which was more pronounced in the films prepared by the non-conventional supercritical solvent impregnation methodology, highlighting the potential of this eco-friendly technique in the production of bioactive films with improved functional properties.

Film packaging materials can house molecules that interact with internal (e.g., food) or external factors (e.g., temperature) and provide dynamic feedback regarding the condition of packed goods (Amin et al., 2022). These intelligent films can respond to environmental changes, such as variation in pH levels, gaseous composition, and microbial activity metabolites (e.g., hydrogen sulfide). The incorporation of these types of molecules in inherently biodegradable matrices (such as NFC and BNC) can be an appealing platform for the mitigation of the environmental footprint imposed by packaging goods. A case in point is the incorporation of poly(sulfobetaine methacrylate) in BNC films (Vilela et al., 2019c). This zwitterionic polymer endows antibacterial activity against *S. aureus* and *E. coli,* while also providing proton conductivity to the material (maximum of 1.5 mS cm^−1^), which can be advantageous for the implementation as sensors to monitor food humidity levels.

### Patches

Nanostructured cellulose-based patches are attractive for a wide range of cosmetic and biomedical applications, including wound healing (Carvalho et al., 2019), drug delivery (Silvestre et al., 2014; Raghav et al., 2021), and skin care (Almeida et al., 2021), due to their inherent biodegradability, biocompatibility, capacity to avoid immunological reactions, and potential to be combined with other components, in particular, bioactive molecules and drugs (Table 3).

**TABLE 3:** Examples of patches produced with cellulosic nanofibrils.

Type of nanofibrils	Other compounds	Methodology	Key properties and applications	Reference(s)
BNC	Lidocaine (4.2 mg cm^−2^) Ibuprofen (1.9 mg cm^−2^)	Diffusion of aqueous or ethanolic solutions in the BNC matrix	Incorporation of hydrophilic or hydrophobic drugs	Trovatti et al. (2011b); Trovatti et al. (2012c)
			Application in drug delivery	
BNC	Diclofenac (1 and 2 mg cm^−2^)	Diffusion of aqueous solutions in the BNC matrix	Fast cumulative release (ca. 90%, after 10 min)	Silva et al. (2014c)
			Permeation studies in the skin (*in vitro*)	
			Application in drug delivery	
BNC	Diclofenac (2.1 mg cm^−2^) Ibuprofen (1.9 mg cm^−2^) Caffeine (8.0 mg cm^−2^) Lidocaine (4.2 mg cm^−2^)	Diffusion of aqueous solutions in the BNC matrix	No noticeable alterations in morphology and release profile after accelerated stability tests	Silva et al. (2020c)
			Application in drug delivery	
BNC	Alginate Chitosan Dexpanthenol (0.32 mg cm^−2^)	Layer-by-layer technology	Modulatory drug release depending on the number of layers of the patch	Fonseca et al. (2020)
			Antibacterial activity: *S. aureus*	
			Non-cytotoxic (HaCaT cells)	
			Promote cell migration	
			Application in wound healing	
BNC	Caffeine (8.0 mg cm^−2^)	Diffusion of aqueous solutions in the BNC matrix	Highly conformable	Silva et al. (2014b)
			Application in skin treatment (cellulite)	
BNC	*E. globulus* leaves hydro-distillation extract (1.0, 1.5, 2.0 and 3.0 μg cm^−2^)	Diffusion of aqueous solutions in the BNC matrix	Antioxidant activity	Almeida et al. (2022)
			Non-cytotoxic (NIH/3T3, HaCaT cells)	
			Minimized senescence of NIH/3T3 cells	
			Application in skin treatment (antiaging)	
BNC	Hyaluronic acid (0.62 mg cm^−2^) Diclofenac (1.56 and 3.12 mg cm^−2^)	Diffusion of aqueous solutions in the BNC matrix	Fast cumulative release (max. 90% after 4 min)	Carvalho et al. (2020)
			Adherent to oral mucosa simulant	
			Non-cytotoxic (HaCaT cells)	
			Application in drug delivery	
BNC	Hyaluronic acid Rutin (14.5 μg cm^−2^)	Micromoulding	The BNC backing layer delays drug release	Fonseca et al. (2021)
			Antioxidant activity	
			Non-cytotoxic (HaCaT cells)	
			Application in drug delivery	
			No adverse skin effects in human participants	
BNC	NSAIDs-based ILs (2.5 mg cm^−2^)	Diffusion of aqueous solutions in the BNC matrix	Increase in drug solubility (up to 100-fold)	Chantereau et al. (2019b)
			Fast cumulative release (ca. 90% after 2 h, for most of the systems)	
			Non-cytotoxic (Raw 264.7 macrophages)	
			Anti-inflammatory activity (in macrophages)	
			Application in drug delivery	
BNC	Phenolic-based ILs (2.5 mg cm^−2^)	Diffusion of aqueous solutions in the BNC matrix	Antioxidant activity	Morais et al. (2019)
			Bolus release, followed by a gradual release up to 24 h	
			Non-cytotoxic (Raw 264.7 macrophages, HaCaT cells)	
			Anti-inflammatory activity	
			Application in skin treatment	
BNC	PMETAC (1.5 and 5)^a^	*in situ* free radical polymerization	Increase in high water uptake capacity	Vilela et al. (2019d)
			Antifungal activity: *C. albicans*	
			Non-cytotoxic (HaCaT cells)	
			Application in the treatment of fungal infections	
BNC	PMGly (1, 2 and 3)^a^ Diclofenac (5 mg cm^−2^)	*in situ* free radical polymerization	pH-dependent drug release	Saïdi et al. (2017)
			Non-cytotoxic (HaCaT cells)	
			Application in drug delivery	
BNC	Vitamin B-based ILs (2.5 mg cm^−2^)	Diffusion of aqueous solutions in the BNC matrix	Increase in vitamin B bioavailability (up to 30.6-fold)	Chantereau et al. (2020)
			Increase in high water uptake capacity	
			Fast cumulative release (at least 66% after 5 min)	
			Non-cytotoxic (HaCaT cells)	
			Application in skin treatment	

^a^ Nominal composition represented as the mass ratio of other compounds in relation to the nanofibrils $\left(W_{other\;compounds}/W_{nanofibrils}\right)$ .

Abbreviations: BNC, bacterial nanocellulose; ILs, ionic liquids; NSAIDs, non-steroidal anti-inflammatory drugs; PMETAC, poly([2-(methacryloyloxy)ethyl]trimethylammonium chloride); PMGly, poly(*N*-methacryloyl glycine).

In the cosmetics industry, cellulose nanofibrils have been widely employed as stabilizers and thickening agents in cream and liquid formulations, as well as moisturizing agents and polymeric matrices (particularly BNC) in facial sheet masks and skin patches (Meftahi et al., 2022). In this latter application, BNC has emerged as an appealing substitute for cotton-based and synthetic polymer-based patches owing to its versatile production with tailored shapes and sizes and capacity to retain a broad range of compounds that can be delivered to the skin in a controlled and sustained manner (Almeida et al., 2021). As such, BNC can act as a carrier of skin-active substances such as caffeine (Silva et al., 2014b), commonly used in cellulite treatment, or plant-based extracts (Almeida et al., 2022), ionic liquids based on phenolic acids (Morais et al., 2019) and complex-B vitamins (Chantereau et al., 2020), which can be used in antiaging formulations. The formulation of these active principles as ionic liquids is aimed at modulating their water solubility and, thus, the resulting release profile. For instance, Chantereau et al. (2020) described an enhancement of the solubility of complex-B vitamins ionic liquids, composed of the vitamin anion and the cholinium cation, compared to the solubility of the original vitamins in water, thereby increasing their bioavailability (up to 30-fold). Moreover, including these active principles in BNC leads to a more complete and faster release of the compounds, which is attractive for short-term applications. BNC has also been reported as the backing layer of microneedles, i.e., arrays of micron-sized needles with 25–2000 μm in height (Fonseca et al., 2019), to support the incorporation of bioactive molecules (e.g., rutin) and delay the release of active pharmaceutical ingredients (APIs) to the skin (delayed cumulative release plateau from 3 to 6.5 h) (Fonseca et al., 2021).

NFC (Raghav et al., 2021) and BNC (Silvestre et al., 2014) based materials can be employed as oral, buccal, or topical drug delivery systems for hydrophilic and hydrophobic APIs. BNC displays its supremacy yet again by facilitating the manufacture of pharmacological patches or other carriers *via* the simple diffusion of APIs aqueous solutions across its three-dimensional porous network. Among the numerous works, we highlight the use of BNC in combination with lidocaine (Trovatti et al., 2011b; 2012c), ibuprofen (Trovatti et al., 2012c), diclofenac (Silva et al., 2014c; Saïdi et al., 2017), diclofenac/hyaluronic acid (Carvalho et al., 2020), and non-steroidal anti-inflammatory drugs (NSAIDs)-based ionic liquids (Chantereau et al., 2019b).

Even when subjected to accelerated testing settings at varying temperatures and relative humidity, BNC patches retain their morphological integrity and release profile (Silva et al., 2020c). In the cases mentioned above, the release of the drugs is essentially governed by their hydrophobic/hydrophilic character diffusion through the 3D network of BNC. However, the modulation of drug release is highly desirable in drug delivery. For instance, Saïdi et al. (2017) modified BNC, under green reaction conditions, with polymers containing amino acid pending moieties to produce patches with pH-responsive behavior.

**FIGURE 4:**
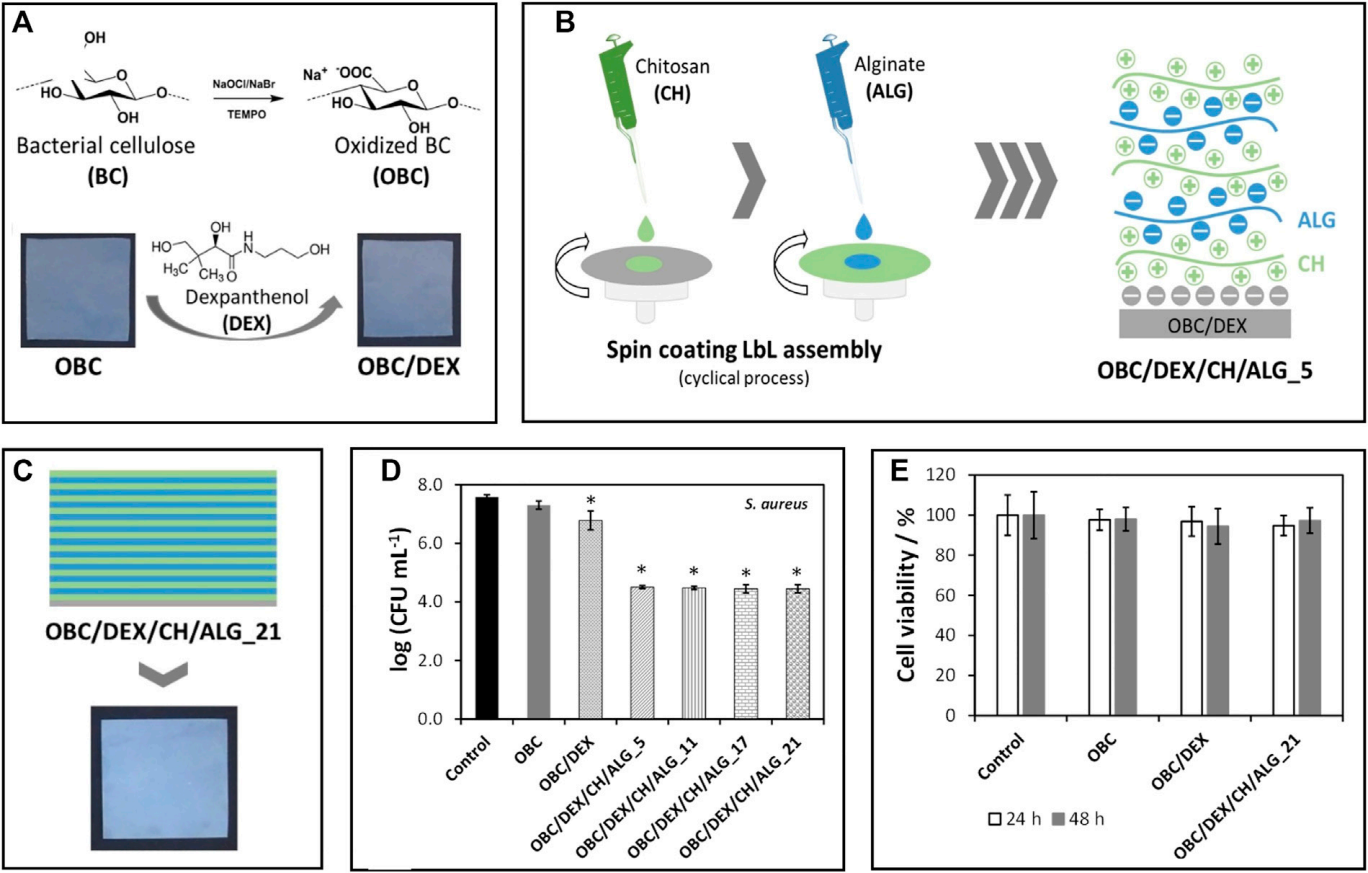
**(A)** Representation of the preparation of TEMPO-mediated oxidated BNC (OBC) and incorporation of dexpanthenol (DEX); **(B)** Scheme of the layer-by-layer spin coating assembly of the patches using alginate and chitosan, and **(C)** Digital photograph of the multilayered patches with 21 layers; Graphical representation of the **(D)** antibacterial activity of the patches against *Staphylococcus aureus* after 24 h of exposure and **(E)** cell viability of HaCaT cells after 24 and 48 h of exposure. Reprinted with permission from (Fonseca et al., 2020). Copyright MDPI, 2020.

In wound healing, BNC is once more the material of choice for most applications due to its high purity and similarity to the extracellular matrix (Carvalho et al., 2019). BNC-based wound dressings provide an adequate moist environment with selective oxygen and water permeability, while simultaneously protecting the injured area from the entry of pathogens and removing the exudates from the site (Carvalho et al., 2019). Additionally, they are simple to detach from the area without inflicting pain or dislodging the newly formed tissue. BNC patches can be functionalized with hemostatic and antimicrobial agents or other bioactive molecules that improve skin cell proliferation and accelerate wound healing. For instance, Fonseca et al. (2020) prepared BNC patches loaded with dexpanthenol, an API used in the treatment of dermatological conditions as a topical protectant and moisturizing agent, and spin-coated the patches with varying layers of alginate and chitosan (Figure 4). The multilayered patches presented antibacterial activity against *S. aureus,* mainly due to the presence of chitosan, a polysaccharide with well-known antimicrobial activity (Wang et al., 2020). They also promoted cell migration and wound closure (*in vitro*) by the inclusion of dexpanthenol in the non-sacrificial BNC matrix. Furthermore, the *in vitro* release profile of the API could be modulated by the number of layers of the polysaccharides, with an extended timeframe of drug delivery as the number of layers increased (Fonseca et al., 2020).

### Other materials

Over the years, researchers have been exploring the exceptional mechanical properties and thermal stability of cellulose nanofibrils for application as reinforcing agents in thermoplastic matrices to improve the properties of the ensuing composites (Shen et al., 2020) (Table 4). BNC has been used as a reinforcement of poly(lactic acid) (PLA) membranes with visible improvements on the mechanical (higher Young’s modulus, compared to neat PLA) and thermal (increase in the initial and maximum degradation temperatures, compared to neat PLA) properties of the materials (Tomé et al., 2011). Unmodified and modified NFC also was used to reinforce poly(ε-caprolactone) (PCL) matrices using a melt-mixing approach, wherein the nanofillers are directly dispersed in the melted polymer and extruded to produce the final nanocomposite materials (Vilela et al., 2019a). The inclusion of NFC modified with cationic latex nanoparticles had no noticeable impact on the composite’s thermal properties (thermal stability up to 335–340°C). However, it improved the mechanical properties (increase in Young’s modulus of the PCL matrices from 41.1 to 52.5 MPa, with the inclusion of only 7.5 wt% of modified NFC) and accelerated the rate of enzymatic degradation of the nanocomposites (from 1.40% for the pure PCL to 1.67–2.21% for the modified nanocomposites, after 10 weeks). This study demonstrated the application of environmentally friendly techniques that improve the compatibility of cellulose nanofibrils with hydrophobic matrices and accelerate the rate at which composites biodegrade. Following a different strategy, Figueiredo et al. (2015b) produced BNC/PCL composites through the addition of the powdered PCL to the culture media, followed by hot pressing of the ensuing membranes, to melt the PCL that was retained inside the porous cellulose network.

**TABLE 4:** Examples of other functional materials produced with cellulosic nanofibrils.

Type of nanofibrils	Other compounds	Methodology	Key properties and applications	Reference(s)
BNC	Graphene oxide (0.025, 0.05 and 0.10)^a^ Phase change materials (0.05 and 0.10)^a^	Solvent casting	Flame retardancy	Pinto et al. (2020b)
			Increased hydrophobicity and hydrophobic behavior	
			Application as thermal/sound insulator materials	
BNC	PCL (2.5, 5, 10 and 20 g L^−1^)	Addition of PCL in the BNC growth media, followed by hot-pressing	Blend of hydrophobic matrices (PCL) and hydrophilic fibers (BNC)	Figueiredo et al. (2015b)
			Improvement of the mechanical properties	
BNC (0.01, 0.04 and 0.06)^b^	PLA	Melt-mixing	Improvement of the thermal stability and mechanical properties	Tomé et al. (2011)
NFC	AgNPs	Electrostatic assembly	Antibacterial activity: *S. aureus; K. pneumoniae*	Martins et al. (2012)
			Application in paper coatings	
NFC	CuNWs (0.01, 0.05, 0.10, 0.20, and 0.50)^a^	Vacuum filtration	Electroconductivity	Pinto et al. (2020a)
			Application in paper coatings	
NFC modified with cationic latex nanoparticles (0.01, 0.05 and 0.075)^b^	PCL	Melt-mixing	Increase compatibility of the fibrils with the matrix due to the cationic latex nanoparticles	Vilela et al. (2019a)
			Enzymatically degradable nanocomposites	
NFC	ZnO NPs	Electrostatic assembly	Antibacterial activity: *S. aureus*; *K. pneumoniae*; *B. cereus*	Martins et al. (2013)
			Application in paper coatings	

^a^ Nominal composition represented as the mass ratio of other compounds in relation to the nanofibrils $\left(W_{other\;compounds}/W_{nanofibrils}\right)$ .

^b^ Nominal composition represented as the mass ratio of nanofibrils in relation to other compounds $\left(W_{nanofibrils}/W_{other\;compounds}\right)$ .

Abbreviations: AgNPs, silver nanoparticles; BNC, bacterial nanocellulose; CuNWs, copper nanowires; NFC, nanofibrillated cellulose; NPs, nanoparticles; PCL, poly(ε-caprolactone); PLA, poly(lactic) acid.

As shown in the previous sections, nanocelluloses can harbor molecules with known functional roles using simple and cost-effective techniques. Nanopapers with inherent conductivity can be prepared by the simple vacuum filtration of NFC suspensions containing copper nanowires (Pinto et al., 2020a), and nanocomposite foams with enhanced fire-retardancy and thermal energy storage capacity may be prepared by solvent casting and freeze-drying of disintegrated BNC nanofibrils, graphene oxide, and phase change materials (Pinto et al., 2020b). Cellulose-based functional coatings prepared *via* the functionalization of nanofibrils with antibacterial moieties [e.g., silver (Martins et al., 2012) and zinc oxide nanoparticles (Martins et al., 2013)] are desirable for many substrates, namely paper materials for packaging solutions. Highly porous nanocellulose materials (e.g., aerogels, cryogels, xerogels, foams, and sponges) are of great interest not only in environmental remediation and catalysis but also in the construction field as thermal and sound insulators (Sun et al., 2021).

Since BNC nanofibrils are analogous to the fibrillar component of the extracellular matrix, nanostructured implantable materials (e.g., injectable hydrogels, tubular grafts, and scaffolds) have been extensively reported in the field of tissue engineering to promote cell regeneration in damaged sites (Carvalho et al., 2019). BNC and NFC nanofibrils can be manipulated to fabricate biomimetic spherical microparticles or microcapsules for cell culture applications (Carvalho et al., 2021b). Cellulosic nanofibrils have also been described as reinforcement additives in the development of bioinks for the 3D printing of hydrogel scaffolds, with application in tissue engineering and 3D cell culture (Teixeira et al., 2022a).

## Exploiting protein fibrils for the production of novel materials

**FIGURE 5:**
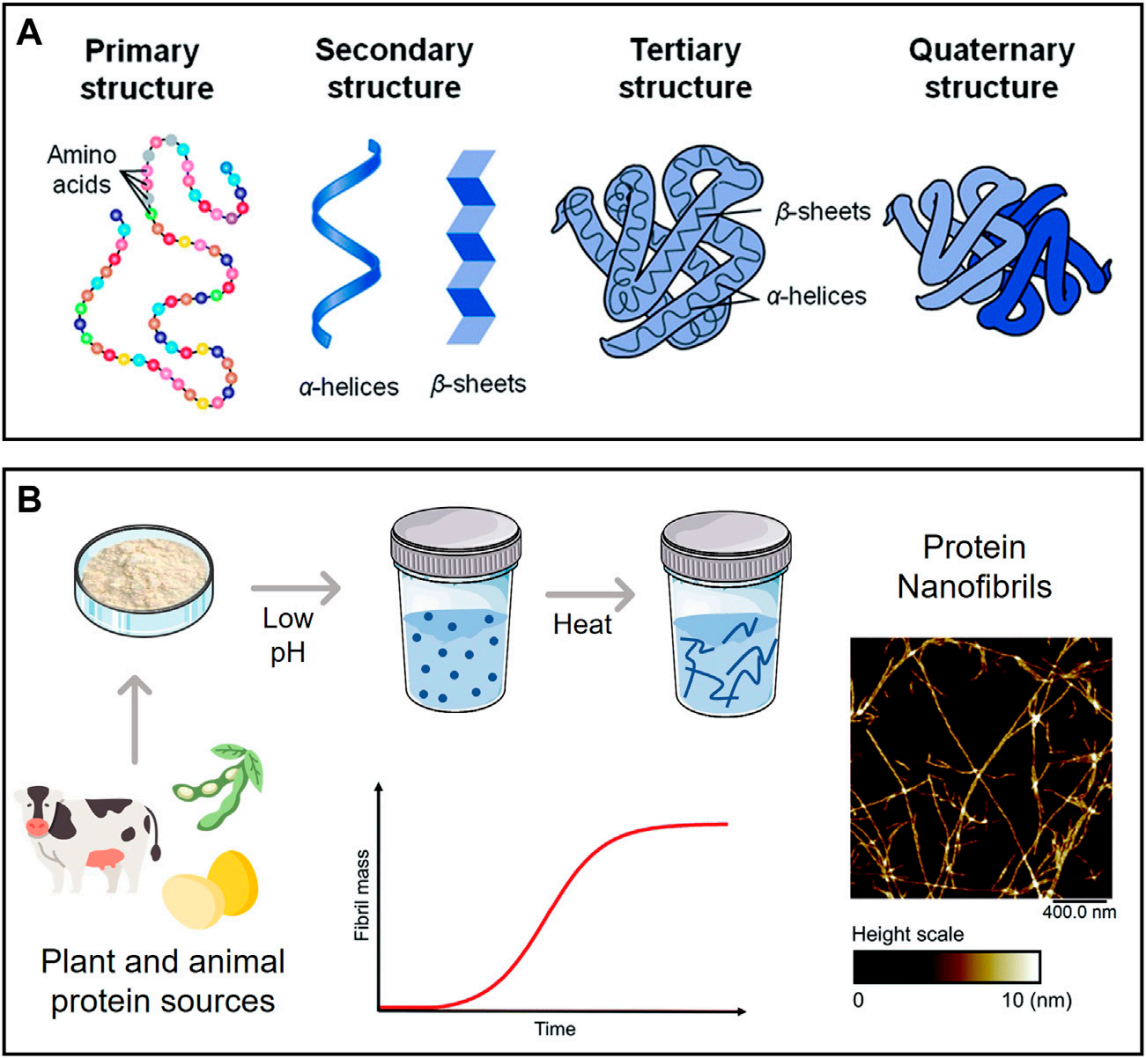
**(A)** Levels of protein organization; **(B)** Schematic representation of a typical process of protein nanofibril production using low pH and elevated temperature, with an illustrative example of their morphology (atomic force microscopy micrograph). Adapted and reprinted with permission from (Silva et al., 2014a). Copyright The Royal Society of Chemistry, 2014; and (Lendel and Solin, 2021). Copyright The Royal Society of Chemistry, 2021.

Proteins (or polypeptides) are among the most prevalent organic macromolecules in living organisms, taking part in important structural and biological roles (Silva et al., 2014a). As illustrated in Figure 5A, they are comprised of a linear sequence of amino acids (primary structure) with a specific local conformation (secondary structure) and a three-dimensional spatial arrangement (tertiary structure). Some proteins can also display a quaternary structure, resulting from the non-covalent interaction between different tertiary structures. These macromolecules can self-assemble into highly ordered amyloid nanofibrils with diameters of 5–10 nm, lengths of several micrometers, and aligned cross-β structures connected by a strong network of hydrogen bonds (Ye et al., 2019). The fibrillation phenomenon is typically linked to the misfolding of soluble proteins and accumulation of agglomerated amyloid nanofibrils (i.e., amyloid plaques) present in neurodegenerative conditions like Parkinson’s and Alzheimer’s (Salahuddin et al., 2021). Several biological proteins are known to self-assemble both *in vivo* and *in vitro* into nanofibrils with non-toxic and functional properties, such as the chorion proteins that protect silkworm eggs and the Pmel17 that is involved in human melanin formation (Ye et al., 2019).

Amyloid nanofibrils can be produced *in vitro* from animal proteins, such as whey protein and hen egg white lysozyme, as study models to examine the principles and mechanisms governing protein self-assembly and fibrillation (Loveday et al., 2017). These protein nanofibrils are formed by partially or entirely unfolding the native structure of the protein, revealing potential amyloidogenic regions in the chain that can assemble into fibrils under specific conditions (Ye et al., 2019). Exposure to low pH (ca. 2) and high temperature is known to promote protein fibrillation (Figure 5B), as is the addition of certain chemicals (e.g., urea, alcohols) and enzymes (e.g., trypsin). The morphology and yield can be adjusted by varying the incubation time, temperature, pH, and ionic strength (Moayedzadeh et al., 2015). Furthermore, microwave irradiation (Carvalho et al., 2016) can shorten the time required to obtain the protein nanofibrils from days to a couple of hours. The use of alternative solvents with benign character as fibrillation agents (e.g., ionic liquids (Silva et al., 2018a) and deep eutectic solvents (Silva et al., 2018c)) is also able to reduce the fibrillation time.

Aside from providing new insights into the pathophysiology triggered by the formation of amyloid nanofibrils, the ability to obtain proteinaceous nanofibrils with exceptional mechanical and thermal stability from animal (e.g., whey protein, hen egg white lysozyme, milk casein) and plant-based (e.g., soy protein isolate, rice glutelin, α-zein) proteins offer an opportunity to use these renewable resources for the development of innovative materials with a high added value (Knowles and Mezzenga, 2016; Lendel and Solin, 2021; Meng et al., 2022). Furthermore, the discovery of functional nanofibrils *in vivo* opens the door to the laboratory synthesis of protein nanofibrils that preserve the inherent biological features of parent proteins, e.g., the antibacterial capabilities of lysozyme (Sarkar et al., 2020), which might be imparted onto the final materials (Table 5).

**TABLE 5:** Examples of functional materials produced with protein-based nanofibrils.

Type of nanofibrils	Other compounds	Methodology	Key properties and applications	Reference(s)
Films				
LNFs (0, 0.01, 0.03, 0.05, 0.10 and 0.15)^a^	Pullulan	Solvent casting	Improvement of the mechanical properties	Silva et al. (2018b)
			Antioxidant activity: ca. 77%, DPPH assay	
			Antibacterial activity: *S. aureus*	
			Application in active food packaging	
LNFs/NFC (2:1, 1:1 and 1:2 mass ratios)		Vacuum filtration	Improvement of the mechanical properties pH-dependent metal sorption capacity (ca. 99%, at pH 11)	Silva et al. (2020a)
			Application in water remediation (removal of Hg^2+^)	
Patches				
LNFs (0, 0.05 and 0.10)^a^	Gelatin Curcumin	Electrospinning	Improvement of the mechanical properties	Carvalho et al. (2022)
			Antioxidant activity: ca. 80%, DPPH assay	
			Reduced bioresorbability rate from 45 to 30–35 days	
			Bust release of curcumin, followed by a constant release for the next 21 days	
			Non-cytotoxic (H9c2 cells, human dermal fibroblasts)	
			Application in tissue regeneration (myocardium)	
LNFs/NFC (1:1 mass ratio)		Vacuum filtration	Antioxidant activity: 76–79%, DPPH assay	Silva et al. (2020b)
			Antibacterial activity: *S. aureus*	
			Non-cytotoxic (L929 fibroblast cells)	
			Promote cell migration	
			Application in wound healing	
Other materials				
LNFs (0.01, 0.05 and 0.10)^a^	Alginate	3D Bioprinting	Non-cytotoxic (HaCaT cells)	Teixeira et al. (2022b)
			Promote cell proliferation for up to 7 days after bioprinting	
			Application in bioink formulation (cell-laden scaffolds)	

^a^ Nominal composition represented as the mass ratio of nanofibrils in relation to other compounds $\left(W_{nanofibrils}/W_{other\;compounds}\right)$ .

Abbreviations: DPPH, 2,2-diphenyl-1-picrylhydrazyl; LNFs, lysozyme nanofibrils; NFC, nanofibrillated cellulose.

In the next sections, relevant examples of the development of new nanomaterials (films, patches and other materials) based on protein nanofibrils will be discussed in detail.

### Films

Protein nanofibrils are incredibly robust, with mechanical strength akin to spider silk and far superior to most biological filaments, and interestingly, also show higher thermochemical stability than their native counterparts (Knowles and Mezzenga, 2016). Nevertheless, the inability of protein nanofibrils to form freestanding films, together with their distinct size and morphology, underline the unique role of protein nanofibrils as nanofillers or templates for the creation of innovative nanocomposite films (Ye et al., 2019). For instance, lysozyme nanofibrils (LNFs) can be combined with the filmogenic polysaccharide pullulan using a simple solvent casting technique (Silva et al., 2018b). The incorporation of LNFs imparted mechanical reinforcement abilities, as demonstrated by the increase in Young’s modulus from 1.69 to 2.50 GPa, and the reduction in the elongation at break from 6.63% to 1.34%, with the addition of 15 wt% of nanofibrils to the pullulan films. Moreover, the inclusion of LNFs endowed antioxidant (ca. 77%, using the DPPH assay) and antimicrobial properties (towards *S. aureus*) to the films, which are highly desirable for application in the food packaging sector.

### Patches

Electrospinning approaches can be employed to obtain other nanomaterials with planar structures, such as patches, from protein suspensions (Lendel and Solin, 2021). For example, amyloid-like bovine serum albumin fibrils were combined with ampicillin to produce electrospun patches that enabled the controlled release of the hydrophilic drug (Kabay et al., 2017). Moreover, even after treatment with solvents prior to the electrospinning process and application of high voltages, the drug retained its antimicrobial action towards *S. aureus* and *E. coli*. In another work, gelatin/LNFs electrospun patches were designed for tissue regeneration, specifically as an implant for infarcted myocardium tissues (Carvalho et al., 2022). The patches displayed excellent mechanical properties (increase in Young’s modulus from 3 to 6 MPa, in wet state) and enhanced antioxidant activity (ca. 80%, using the DPPH assay), ascribed to the reinforcement with the protein nanofibrils. Furthermore, the materials had an increased bioresorbability rate compared to patches comprised only of gelatin (reduction in 10–15 days), highlighting the immense potential of LNFs as functional reinforcements.

### Other materials

**FIGURE 6:**
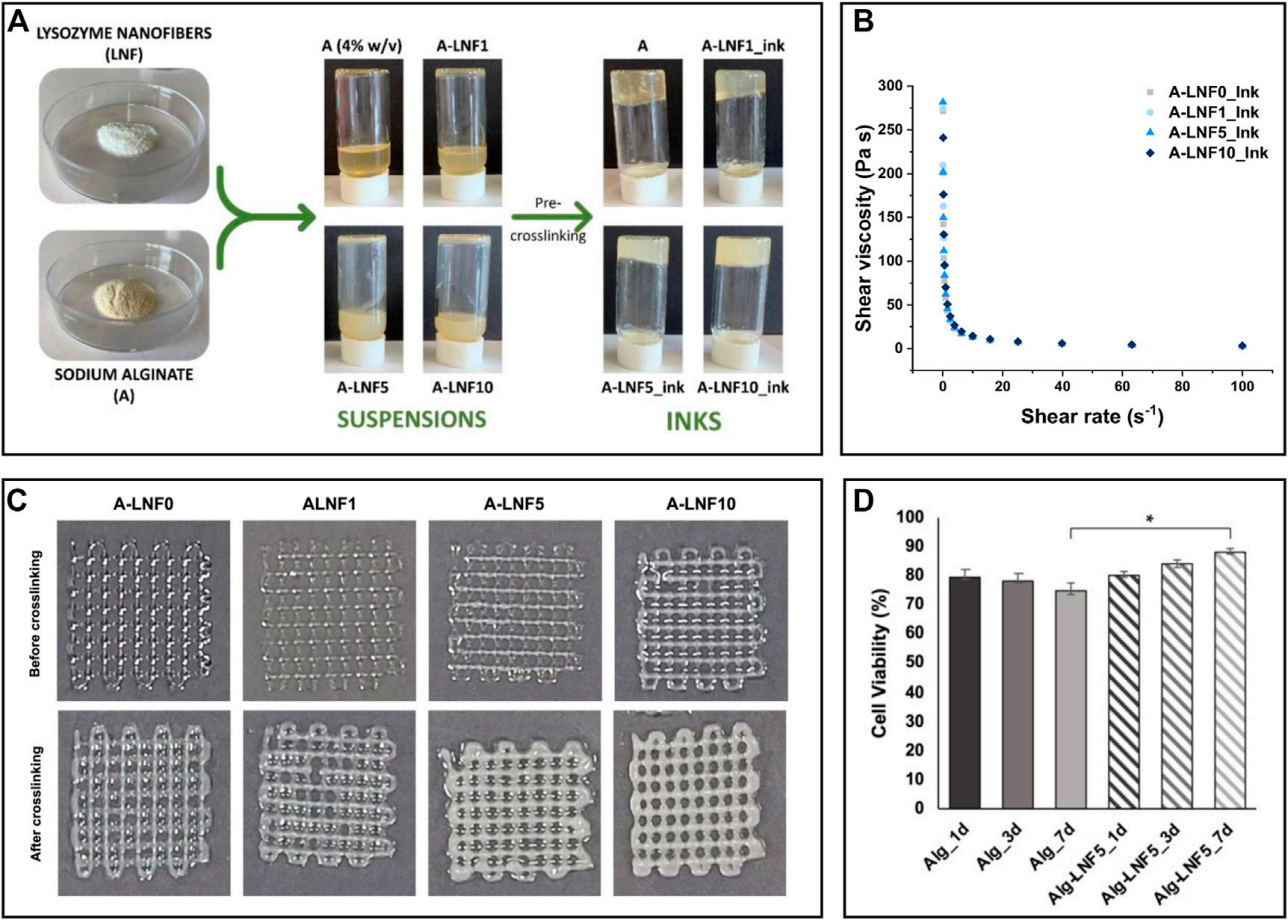
**(A)** Illustration of the preparation of the alginate and lysozyme nanofibrils (LNFs) bioinks; **(B)** Graphical representation of the shear viscosity of the inks with different contents of LNFs; **(C)** Optical micrographs of the printed scaffolds (2 layers) before and after crosslinking with CaCl_2_; **(D)** Graphical representation of the cell viability of HaCaT cells incorporated in the scaffolds after 1, 3 and 7 days of bioprinting. Reproduced with permission from (Teixeira et al., 2022b). Copyright MDPI, 2022.

Owing to the increase in viscosity during fibrillation, which is related to the interfibrillar interactions facilitated by the high aspect ratio of the nanofibrils, protein nanofibril dispersions demonstrate impressive gelation capabilities, even at low concentrations (Lendel and Solin, 2021). This ability is responsible for the formation of hydro/aero-gels with high stability, which find applicability in the biomedical field as scaffolds for cell and tissue growth and bioink formulations used in 3D printing technology (Veiga et al., 2021). In this realm, composite hydrogels of LNFs and alginate were produced to formulate bioinks for extrusion-based 3D bioprinting (Figure 6A) (Teixeira et al., 2022b). The reinforcement of the alginate matrix with the protein nanofibrils was responsible for improving the shear-thinning behavior, which is crucial in the printing process (Figures 6B,C). As a proof of concept, the gels were loaded with immortalized human keratinocytes (HaCaT cells). After printing the scaffold structures, the suitability of the hydrogels was evaluated *via* cell viability. Not only were the structures able to maintain the cells alive during the studied time (7 days), but the structures printed with the bioinks containing LNFs showed enhanced cell proliferation (cell viability of ca. 88%) compared to the cell-laden hydrogels without the protein nanofibrils (cell viability of 75%), highlighting their bioactive role (Figure 6D).

The addition of protein nanofibrils is also known to alter the mechanical behavior of thermoplastic matrices. For instance, the blend of bovine insulin nanofibrils with poly(vinyl alcohol) resulted in increased stiffness of the films compared with the addition of the same amount (1 wt%) of non-fibrillar bovine insulin (Rao et al., 2012). The increase of other mechanical properties, such as elongation at break and elastic modulus, has also been reported with the blend of LNFs with poly (lactic acid) (Byrne et al., 2011) and a silicone elastomer (Oppenheim et al., 2010), respectively.

## The partnership between nanocellulose and protein fibrils

The use of cellulose nanofibers or protein fibrils in combination with other (bio)polymers or bioactive molecules results in nanostructured materials with promising physicochemical properties and functionalities, which can take the form of membranes, films, patches, and other materials, as illustrated in the works mentioned above (Tables 1–5). Given the tremendous potential of cellulose and protein nanofibrils on the design of new functional materials, their assembly will certainly result in fibrillar materials with unique properties. Nonetheless, as far as we could gather, only three publications to date dealing with the joint use of nanocellulose and protein nanofibrils in advanced material production, namely in films and aerogels with application in water remediation (Silva et al., 2020a; Sorriaux et al., 2021) and patches for wound healing (Silva et al., 2020b), have been reported (Table 5).

Silva et al. (2020a) described for the first time the preparation of NFC/LNFs biobased films using a simple methodology of vacuum filtration of the water-based suspensions of nanocellulose (obtained from softwood) and protein nanofibrils (extracted from hen egg white). The dual nanofibrillar films exhibited superior mechanical properties compared to neat NFC films (which already have remarkable mechanical properties), namely an increase up to 2 GPa of the Young’s modulus and a concomitant decrease in the elongation at break. These results highlighted the structural reinforcement role of the lysozyme nanofibrils in the system, possibly due to the interactions between the hydroxyl and carboxyl groups of the nanocellulose fibrils and the amide groups of the LNFs. The adequacy of the NFC/LNFs films as biosorbents was evaluated in mercury-contaminated ultrapure and spring waters. After 24 h of contact at pH 11 (close to the isoelectric point of the lysozyme), the removal effectiveness reached a maximum of 99% and a residual concentration below the threshold value in waters intended for human consumption. The presence of amino acid side chains with multiple binding sites plays a crucial role in the adsorption of the Hg^2+^ ions.

In the same field, β-lactoglobulin nanofibrils were combined with polydopamine-coated cellulose nanofibrils (NFC) and crosslinked *via* periodate oxidation to produce biosorbent aerogels (Sorriaux et al., 2021). The adsorption capability of the aerogels was evaluated in water contaminated with an assortment of pollutants (e.g., dyes, pesticide/pharmaceutical agents, and heavy metal ions), with good efficiencies and fast adsorption rates in the removal of crystal violet dye (93%, 30 min), bisphenol A (92%, 5 min) and Pb^2+^ ions (95%, 5 min), specifically. In this case, the adsorption is facilitated by the presence of various functional groups (e.g., catechols, quinones amines, and aromatic moieties) in the polydopamine functional coating.

**FIGURE 7:**
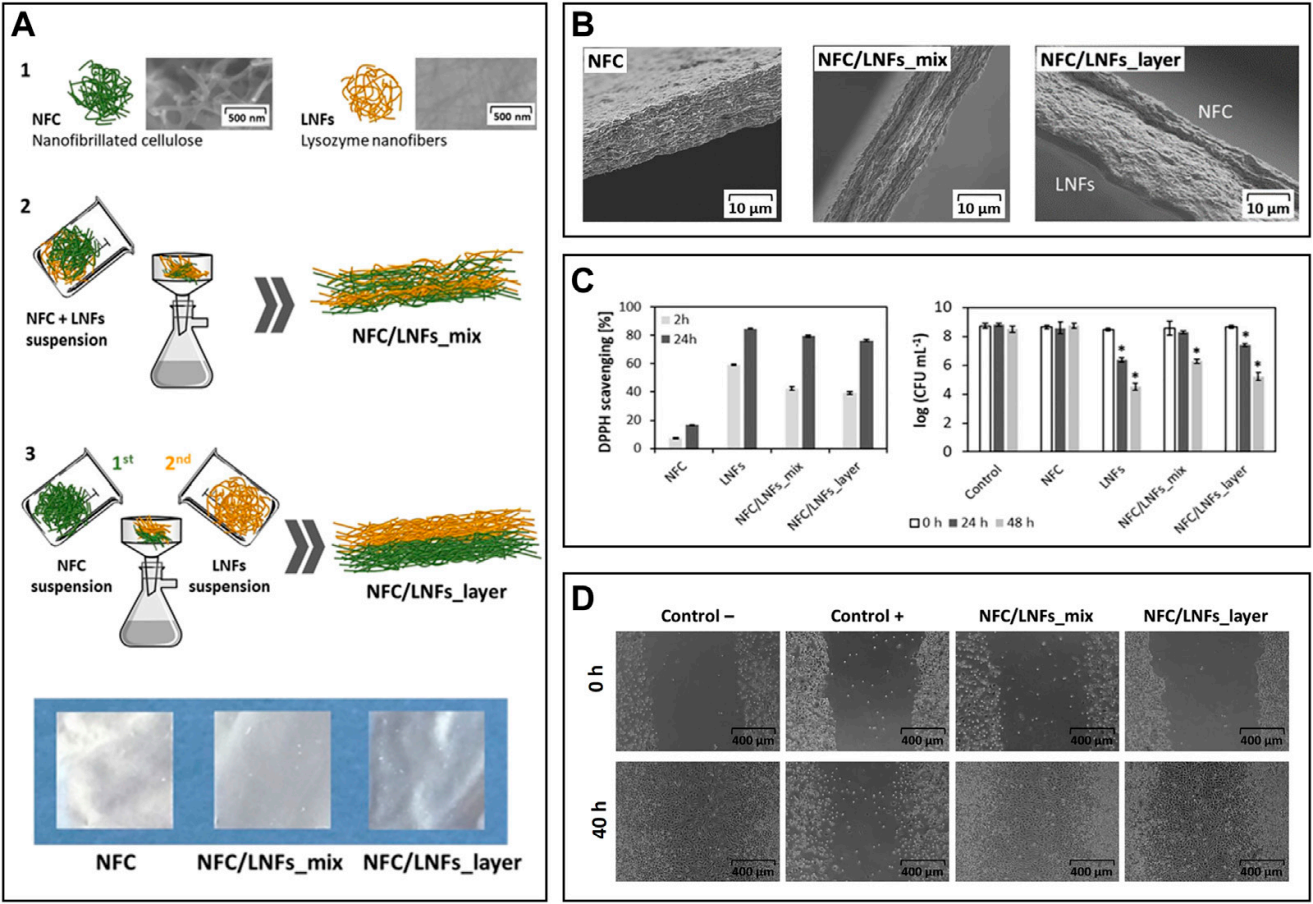
**(A)** Overview of the constituents (1) and the preparation of the nanocellulose (herein represented as NFC) and lysozyme nanofibrils (LNFs) patches *via* vacuum filtration by direct mixing of the suspensions (2) or using a layered approach (3), and digital photographs of the dry patches; **(B)** Scanning electron microscopy micrographs of the cross-section of the patches; **(C)** *In vitro* antioxidant activity (left) and antimicrobial activity (right) of the functional patches; **(D)** Optical micrographs of the scratch assay of the fibroblast cells after 40 h of exposure to both patches. Reproduced with permission from (Silva et al., 2020b). Copyright Elsevier, 2020.

The combination of NFC and LNFs was also evaluated in the preparation of nanofibrillated patches for wound healing following two different approaches, *viz.*, patches produced *via* vacuum filtration of the mixed NFC and LNF dispersions *versus* ones obtained through the sequential filtration of NFC and LNFs, respectively (Figure 7A) (Silva et al., 2020b). Scanning electron microscopy analysis revealed the excellent compatibility between NFC and LNF in the mixed nanofibrils patch and in the two distinct layers in the patch obtained by sequential deposition of NFC and LNF (Figure 7B). The mechanical properties differed due to the varying layouts. Compared to the pure NFC patch, the blended nanofibrils patch displayed an increase in Young’s modulus, as expected, from 4.4 to 6.7 GPa. This trend, however, was not mirrored in the layered nanofibrils patch, which exhibited a lower Young’s modulus than neat NFC (3.7 GPa), most likely due to the exclusive establishment of interfacial interactions between the functional groups of both nanofibrils. The inclusion of proteinaceous nanofibrils provided the patches with good UV-barrier properties, high antioxidant activity (up to 79.5%, using the DPPH assay), and antimicrobial activity against *S. aureus* (up to 3.5-log CFU mL^−1^ reduction), which was slightly higher in the layered patch due to the bacterium’s direct contact with the LNFs side (Figure 7C). Both NFC/LNFs patches were biocompatible toward the L929 fibroblast cell line and, in contrast to the pure NFC patch, promoted cell adhesion with high viability values (Figure 7C). The *in vitro* wound healing assay exhibited good migratory capacity of the cells on the surface of the patches, resulting in nearly complete occlusion of the simulated wound (Figure 7D). These findings point to the potential of the dual nanofibrils patches in wound healing improvement.

## Conclusion and future perspectives

In this review, we outlined the properties of cellulose and protein (amyloid) nanofibrils in the development of ecofriendly and sustainable advanced materials, namely membranes, films, and patches, among others (Tables 1–5). Owing to their excellent mechanical strength, these biobased nanofibrils are frequently used as reinforcement agents by compounding with thermoplastics such as PLA (Tomé et al., 2011) and PCL (Figueiredo et al., 2015b; Vilela et al., 2019a), or through combination with biopolymeric matrices like pullulan (Trovatti et al., 2012a; Trovatti et al., 2012b), resulting in materials with improved mechanical and thermal properties.

Apart from the reinforcement role, the remarkable physicochemical properties of nanofibrils (e.g., high water absorption capacity, high surface area, biocompatibility and biodegradability) are being exploited in the development of advanced functional materials (Tables 1–4). BNC is particularly interesting for some applications since its membranes are simple to obtain in tailored shapes and sizes (*in situ* moldability) to better suit the target application. Combining BNC with bioactive molecules or polymers with different functionalities can produce materials for widely different applications, which explains the deluge of works reported using this nanocellulosic form.

Overall, materials produced with BNC and NFC have received much attention in a variety of emerging fields, including fuel cell components (Gadim et al., 2014; Gadim et al., 2016; Vilela et al., 2016; Gadim et al., 2017; Vilela et al., 2017; Vilela et al., 2018b; Vilela et al., 2020a; Vilela et al., 2020c; Vilela et al., 2020b), water remediation (Vilela et al., 2019b), active food packaging (Vilela et al., 2019c; Moreirinha et al., 2020; Bastante et al., 2021), skin treatment (Silva et al., 2014b; Morais et al., 2019; Chantereau et al., 2020; Almeida et al., 2022), wound healing (Fonseca et al., 2020) and drug delivery (Trovatti et al., 2011b; Trovatti et al., 2012c; Silva et al., 2014c; Silva et al., 2020c; Saïdi et al., 2017; Chantereau et al., 2019b; Carvalho et al., 2020; Fonseca et al., 2021).

Contrary to cellulose nanofibrils, protein amyloid nanofibrils, apart from their excellent mechanical properties, biodegradability and biocompatibility, also carry the functional groups of their native amino acid chains, avoiding an additional step in modification. Given so, the ability to hold intrinsic functional properties, paired with their high surface area and capacity to mimic non-cellular components, is attracting tremendous interest in the use of protein nanofibrils as functional reinforcements for the development of films, patches and other materials (Table 5) with application in areas like active food packaging (Silva et al., 2018b), bioinks formulation (Teixeira et al., 2022b) and tissue regeneration (Carvalho et al., 2022).

Moreover, the design of dual-fibrillar systems comprising both nanocellulose and protein nanofibrils seems to be a promising approach, still in its infancy. Regardless, their successful partnership is notorious, particularly in the formulation of patches for biomedical treatments (Silva et al., 2020b) and in the development of films (Silva et al., 2020a) and aerogels (Sorriaux et al., 2021) for environmental remediation strategies. In these studies, only NFC and two protein nanofibrils (lysozyme and β-lactoglobulin) have been used; therefore, plenty of materials can be foreseen by the combination of other nanocelluloses with nanofibrils obtained from other protein sources.

Even though the transition to more sustainable biopolymeric nanofibrillated materials remains limited to the establishment of straightforward, economically viable, and speedier methods for nanofibril production, there is a clear potential for economic expansion in this field. The global nanocellulose market is estimated to reach USD 1,053.09 million by 2027, with a compound annual growth rate (CAGR) of 19.9% throughout the forecast period (2020–2027), demonstrating the clear interest in cellulosic nanofibrils (Fortune Business Insights, 2021). The functional protein industry is still primarily driven by uses for animal feed, nutraceuticals, and food and beverage supplementation (Markets and Markets, 2021). However, the growing interest in this research field over the past few years might be a driving factor towards the development of functional materials (Ye et al., 2019; Daniloski et al., 2021; Lendel and Solin, 2021; Peydayesh and Mezzenga, 2021; Vinayagam et al., 2022). Protein nanofibrils have significant advantageous characteristics and can be valuable building blocks in the creation of sophisticated nanostructured materials with intrinsic functional properties. Despite the significant progress that remains to be accomplished in this field, we foresee a growing interest in the use of nanocellulosic and protein nanofibrils, as well as their blends, as we begin to witness the merits of this partnership (Silva et al., 2020a; Silva et al., 2020b; Sorriaux et al., 2021).
